# Supplementary material for: Revealing electronic correlations in YNi2B2C using photoemission spectroscopy
Source: Commun Phys. 2025 Jun 17;8(1):256. doi: 10.1038/s42005-025-02180-4 (PMC12173938; doi:10.1038/s42005-025-02180-4)
Supplement: Supplementary file 1 — Supplementary Information [file 42005_2025_2180_MOESM1_ESM.pdf]

Supplementary Information for "Revealing electronic correlations in YNi_2B_2C using photoemission spectroscopy"

Aki Pulkkinen,[1,2] Geoffroy Kremer,[2,3] Vladimir N. Strocov,[4] Frank Weber,[5] Ján Minár,[1] and Claude Monney[2]

[1] *New Technologies-Research Centre, University of West Bohemia, 30100 Plzeň, Czech Republic*

[2] *Département de Physique and Fribourg Center for Nanomaterials, Université de Fribourg, CH-1700 Fribourg, Switzerland*

[3] *Institut Jean Lamour, UMR 7198, CNRS-Université de Lorraine, Campus ARTEM, 2 allée André Guinier, BP 50840, 54011 Nancy, France*

[4] *Paul Scherrer Institut, Swiss Light Source, 5232 Villigen PSI, Switzerland*

[5] *Institute for Quantum Materials and Technologies, Karlsruhe Institute of Technology, Kaiserstr. 12, D-76131 Karlsruhe, Germany*

SUPPLEMENTARY NOTE 1

Comparison of FP-LAPW and FP-KKR band structures

For benchmarking our FP-KKR electronic structure, we make a comparison to another, well established all-electron method, the full potential linearized augmented plane wave (FP-LAPW) method implemented in the Elk software. The band structures calculated with the PBE exchage-correlation functional in the energy range from -11 eV to 1 eV are presented in Fig. S1, and show an excellent agreement between the two methods.

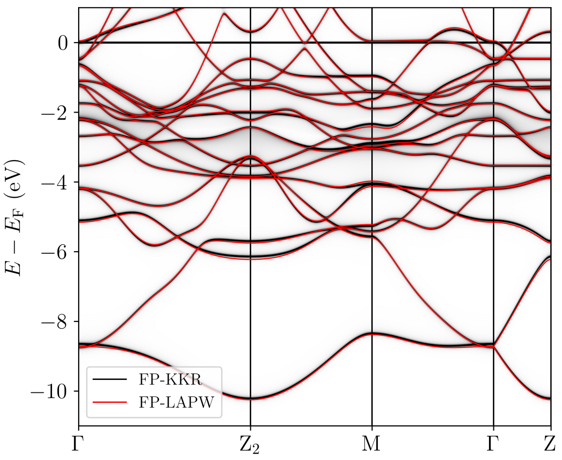

FIG. S1. Comparison of YNi_2B_2C band structures calculated within the GGA using the full potential KKR method (black lines) and the FP-LAPW method (red lines).

SUPPLEMENTARY NOTE 2

Reducing the inelastic background of the experimental ARPES data

The inelastic background in the experimental ARPES data was reduced by applying a Gaussian filter to broaden the features and subtracting the broadened spectrum from the original. We define a region of interest (ROI) where we wish to sharpen the spectral features, which was chosen from $-3.5\,\mathrm{eV}$ to the Fermi energy. First, a constant background value is subtracted from the experimental data to reduce the overall signal level. Second, the ARPES data is broadened by applying a Gaussian filter along the momentum axis with a standard deviation σ_k. The broadened map S_{σ_k} is subtracted from the original map S with a weight factor f_1:

$$S_1 = S - f_1 S_{\sigma_k}.$$

The factor f_1 is determined heuristically by gradually increasing f_1 and monitoring that the amount of negative values in S_1 does not exceed a predefined level. A similar procedure is repeated to S_1 along the energy axis with a standard deviation σ_E and a weight factor f_2. The subtractions introduce negative values in the low-intensity regions, that are eventually set to zero. Figure S2 demonstrates the effect of the background subtraction procedure and shows that it is able to reduce the broad background significantly and allows to identify bands more clearly in the areas with strong inelastic background.

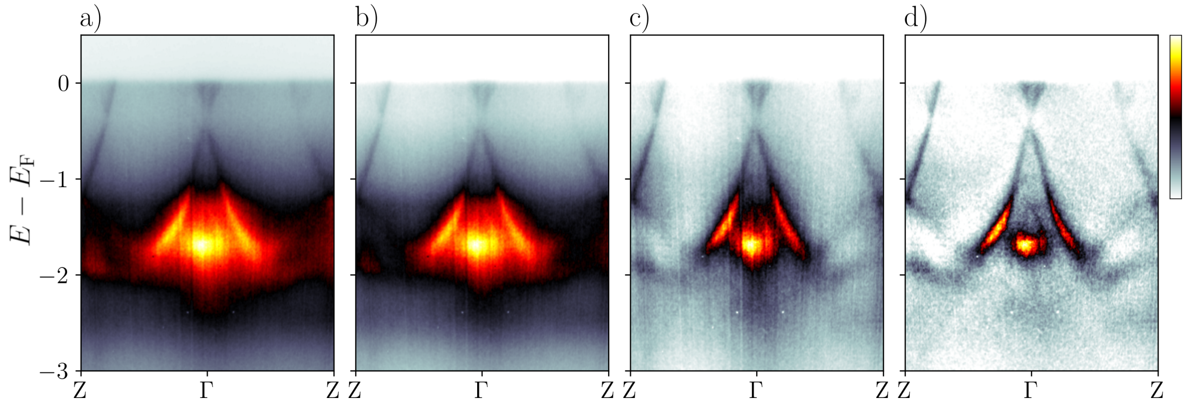

FIG. S2. The effect of the background subtraction procedure on the experimental data along the Z–Γ–Z direction. a) Original (k, E) map. b) Constant background subtracted. c) k-broadened map subtracted. d) E-broadened map subtracted.

SUPPLEMENTARY NOTE 3

Matching the perpendicular momentum k_z in theory and experiment

The momentum component perpendicular to the surface is not conserved in the photoemission process and its value depends *i.a.* on the work function of the surface and the inner potential of the material. It is therefore important to make sure that the k_z sampled in the calculations correspond to the one in experiment. This is a prerequisite for making reliable comparisons with experiment and theory. To determine which perpendicular momentum value k_z is sampled at a given photon energy in the one-step model calculations, we have calculated a $(k_\mathrm{x}, k_\mathrm{z})$ map in a plane that crosses the M point. The M point is chosen as the sampled point, because the large intensity variation at this point is a clear indicator of the k_z. The results are presented in Fig. S3. We conclude that the $k_z = 23.0\,$r.l.u. sampled at photon energy $693\,$eV is reached in the one-step model calculations at photon energy $709\,$eV.

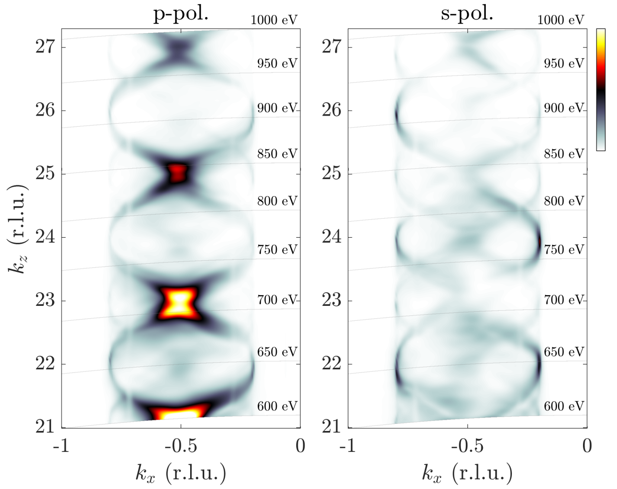

k_z (r.l.u.)	E_phot (eV)
22.0	648
22.5	678
23.0	709
23.5	740
24.0	772
24.5	805
25.0	839
25.5	873
26.0	908

FIG. S3. Left: One-step model (k_x, k_z) maps calculated with p- and s-polarized photons in a plane that crosses the M point. Right: Coordinates of high-symmetry points along the k_z direction and the corresponding photon energies.

SUPPLEMENTARY NOTE 4

Site-resolved band structure

The FP-KKR GGA site-resolved band structure of YNi_2B_2C is presented in Fig. S4 in the energy range from -11 eV to 1 eV. The bands just above the Fermi level originate from Y and Ni. The energy range from -4 eV to the Fermi level mainly consists of Ni states, and the rest of the bands down to -11 eV have mixed character from B, Ni, and C.

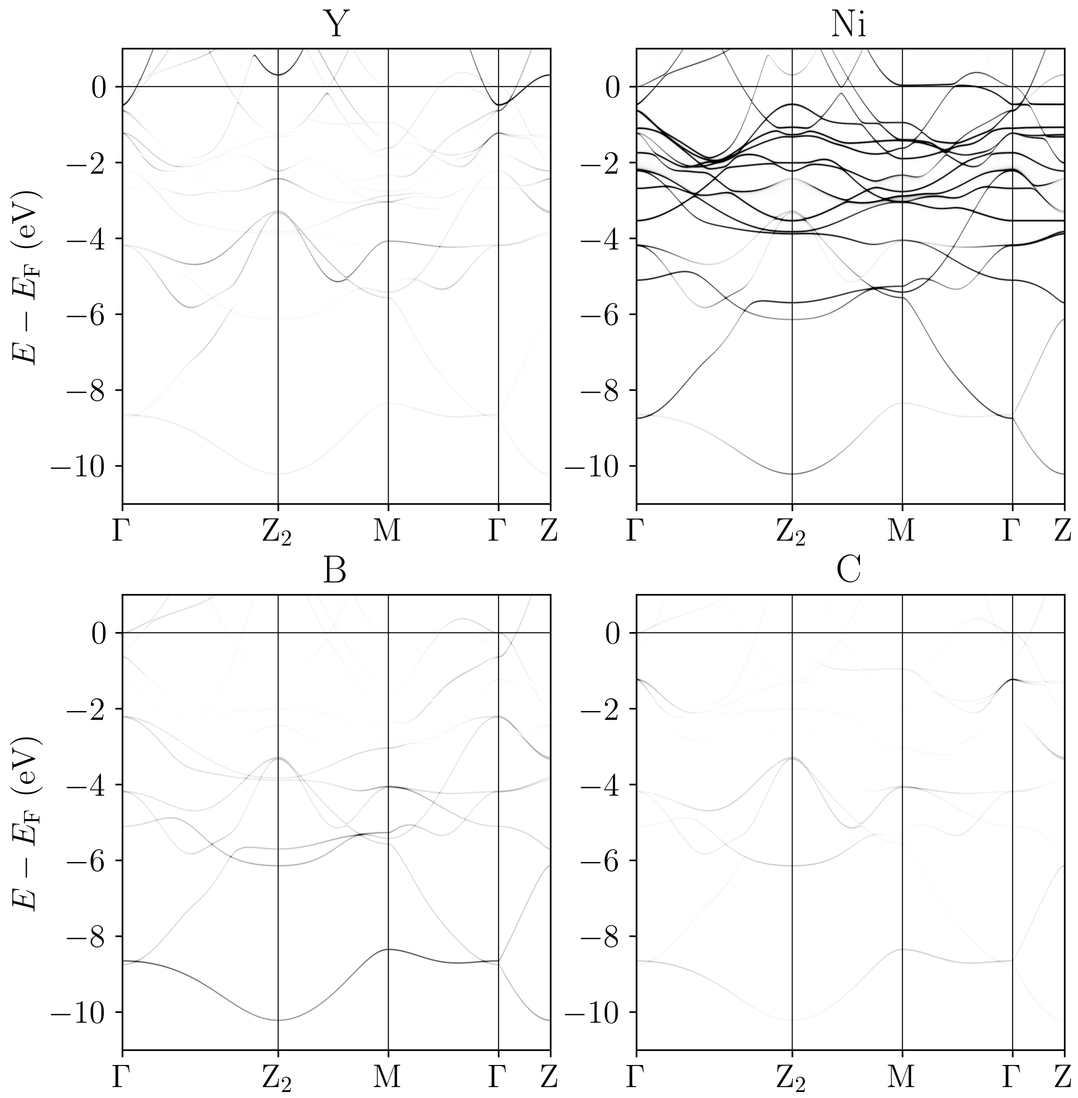

FIG. S4. FP-KKR GGA band structure of YNi_2B_2C projected on the Y, Ni, B and C sites.

SUPPLEMENTARY NOTE 5

Comparison of one-step model calculation and Bloch spectral function

Figure S5 shows comparisons between one-step model calculations and the corresponding Bloch spectral function along the same momentum path with DFT-GGA and DFT+DMFT. The one-step model calculation, taking into account the photoemission matrix elements, makes it possible to identify the bands with high intensity in the ARPES experiments.

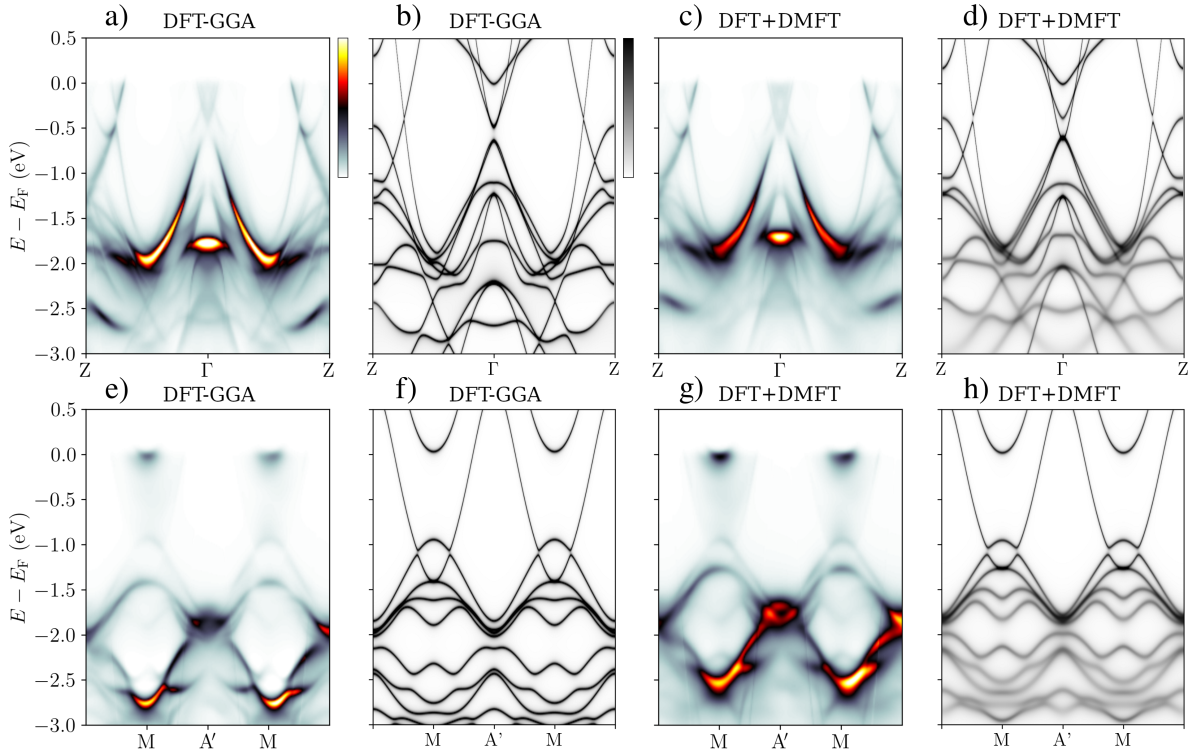

FIG. S5. a)-d) One-step model calculations with DFT-GGA and DFT+DMFT compared to corresponding Bloch spectral function calculations in the Z-Γ-Z direction. e)-h) Similar comparison for the M-A'-M direction.
